# Age-structured Jolly-Seber model expands inference and improves parameter estimation from capture-recapture data

**DOI:** 10.1371/journal.pone.0252748

**Published:** 2021-06-09

**Authors:** Nathan J. Hostetter, Nicholas J. Lunn, Evan S. Richardson, Eric V. Regehr, Sarah J. Converse

**Affiliations:** 1 Washington Cooperative Fish and Wildlife Research Unit, School of Aquatic and Fishery Sciences, University of Washington, Seattle, Washington, United States of America; 2 Wildlife Research Division, Science and Technology Branch, Environment and Climate Change Canada, Gatineau, Canada; 3 Polar Science Center, Applied Physics Laboratory, University of Washington, Seattle, Washington, United States of America; 4 U.S. Geological Survey, Washington Cooperative Fish and Wildlife Research Unit, School of Environmental and Forest Sciences & School of Aquatic and Fishery Sciences, University of Washington, Seattle, Washington, United States of America; Wildlife Conservation Society, INDIA

## Abstract

Understanding the influence of individual attributes on demographic processes is a key objective of wildlife population studies. Capture-recapture and age data are commonly collected to investigate hypotheses about survival, reproduction, and viability. We present a novel age-structured Jolly-Seber model that incorporates age and capture-recapture data to provide comprehensive information on population dynamics, including abundance, age-dependent survival, recruitment, age structure, and population growth rates. We applied our model to a multi-year capture-recapture study of polar bears (*Ursus maritimus*) in western Hudson Bay, Canada (2012–2018), where management and conservation require a detailed understanding of how polar bears respond to climate change and other factors. In simulation studies, the age-structured Jolly-Seber model improved precision of survival, recruitment, and annual abundance estimates relative to standard Jolly-Seber models that omit age information. Furthermore, incorporating age information improved precision of population growth rates, increased power to detect trends in abundance, and allowed direct estimation of age-dependent survival and changes in annual age structure. Our case study provided detailed evidence for senescence in polar bear survival. Median survival estimates were lower (<0.95) for individuals aged <5 years, remained high (>0.95) for individuals aged 7–22 years, and subsequently declined to near zero for individuals >30 years. We also detected cascading effects of large recruitment classes on population age structure, which created major shifts in age structure when these classes entered the population and then again when they reached prime breeding ages (10–15 years old). Overall, age-structured Jolly-Seber models provide a flexible means to investigate ecological and evolutionary processes that shape populations (e.g., via senescence, life expectancy, and lifetime reproductive success) while improving our ability to investigate population dynamics and forecast population changes from capture-recapture data.

## Introduction

Age structure affects population dynamics and how populations respond to environmental change [1]. For many organisms, age is an important factor determining an individual’s contributions to population growth [2]. At the population level, differences in age structure can result in divergent population trajectories even if total abundances are comparable and populations are exposed to similar environmental conditions. Knowledge of age structure, age-specific demographic rates, age-specific abundance, and how these factors interact through time is important for detailed assessments of population dynamics, viability, and the consequences of environmental variation and management actions [3–6]. Although shifting age structures influence population dynamics and may cause substantial deviations from asymptotic projections, age structure and changes in age structure through time are rarely estimated in studies of free-ranging wildlife [3, 6].

Capture-recapture methods provide a robust framework to estimate demographic processes and incorporate auxiliary information to improve inferences [7]. Collection of age data or its correlates (e.g., size or length) is common in capture-recapture studies (e.g., [8] and citations therein). The importance of age on demographic rates has led to a variety of single- and multi-state capture-recapture models that condition on initial capture (i.e., estimate demographic rates, not abundance) to investigate age-dependent survival in terrestrial and aquatic ecosystems [5, 7, 9–11]. Inference is generally limited to estimates of demographic rates for the marked subset of a population. Methods to incorporate age- or age-related data in open-population capture-recapture models that do not condition on first capture and can jointly estimate survival, recruitment, and abundance (e.g., Jolly-Seber models), however, are more limited and lack a unifying framework. Efforts to include age or age-like effects in Jolly-Seber (JS) models include population reconstructions [12], estimating residency time in stopover duration models [13–16], and modified open-population models with Horvitz-Thompson estimators for abundance [8]. While each of these approaches address study-specific challenges, they involve analyses detached from the JS framework [8, 12], require study durations that are greater than the lifespan of the study species [14, 15], or involve modeling demographic processes prior to the study to account for individuals born before the first occasion [15]. These challenges are particularly limiting for studies of long-lived species, which are often of high conservation concern and a regular focus of long-term capture-recapture studies to monitor abundance and demographic rates [17, 18].

Here, we develop a novel and generalizable age-structured JS open population model to jointly estimate age-specific demographic rates, abundance, population age structure, annual changes in age structure, and recruitment from capture-recapture and age data. Our approach integrates six processes into one state-space JS model [19–21]: (1) age structure in the first year of the study (i.e., year 1), (2) recruitment, (3) aging, (4) survival, (5) abundance, and (6) imperfect detection from capture-recapture data. This age-structured JS approach is straightforward to generalize and can be applied to any species where capture-recapture and age data (or correlates thereof) are collected, including when some observed individuals are missing age data. The novelty of our approach lies in treating individual age in year 1 as a random variable, where 0 is not yet born and values > 0 indicate initial age (i.e., born prior to the study). After occasion 1, the traditional JS recruitment process describes new births, and individuals age deterministically after birth. Modeling year-1 age-structure, rather than birth and survival processes for the lifespan of the species prior to the study [15], is particularly beneficial for studies of long-lived species because modeling decades of demographic processes prior to the study and the associated assumptions are no longer required. This extension is also wholly contained within the family of JS models [e.g., 16, 19, 21, 22], which provides opportunities to incorporate covariates, individual heterogeneity, and alternative parameterizations [21, 22] while creating new possibilities to incorporate age data, growth models, and investigation of age-related ecological hypotheses that are common in conditional-on-first-capture capture-recapture studies [5, 7, 9–11, 23] but generally missing in studies utilizing JS models. Further, because it is formulated as an individual-based model, our approach extends to studies with incomplete age data, correlates of age, and a variety of age-dependent demographic studies including evolutionary and life history analyses (e.g., senescence, life expectancy, reproductive success), recruitment, changes in age structure through time, and population viability analyses.

We begin by describing a state-space JS model using the Schwarz and Arnason superpopulation formulation [24, 25] (hereafter JS model) and then extend the JS model to incorporate age structure. We use simulation to validate that the age-structured JS model (1) returns unbiased parameter estimates, (2) improves parameter precision relative to the JS model without age data, and (3) extends inference from the JS model to include unbiased estimates of age-specific demographic processes.

We applied the age-structured JS model to a case study investigating polar bear (*Ursus maritimus*) recruitment, abundance, age-dependent survival, and age structure in western Hudson Bay, Canada (hereafter WHB). Improved estimation methods from limited data are particularly relevant for managing polar bears and other difficult-to-study species in rapidly changing environments [26]. Although capture-recapture studies are widely used to estimate polar bear vital rates and abundance [27–29], age data provide information to investigate hypotheses on age-class specific survival (e.g., subadult vs adult) [28, 29], senescence [30–32], and age structure [33]. However, in many cases the precision of demographic parameter estimates is low and some parameters, particularly abundance, exhibit unrealistic fluctuations from year to year [27–30]. Integrating capture-recapture and age data via an age-structured JS model provides new opportunities to address these challenges while also addressing the influence of age structure on population dynamics.

## Materials and methods

### Jolly-Seber superpopulation formulation

We follow the methods of [25] for Bayesian analysis of the JS superpopulation model (i.e., state-space formulation using data augmentation; see also [34]), where the superpopulation is defined as the group of animals that are part of the population at any point during the study period. Data augmentation produces a dataset of *M* individuals that are allocated among *K* study occasions according to a multinomial process for entry probabilities. Each individual in the augmented data set has an inclusion parameter *w*_*i*_, where *w*_*i*_ = 1 if the individual is part of the superpopulation and 0 otherwise. We assume

$$w_{i}\sim Bernoulli(\psi )$$ (1)

where *ψ* is the probability an individual in the augmented data set is part of the superpopulation. Note, *w*_*i*_ = 1 is known for any observed individual. Individuals recruit into the superpopulation during one of *K* occasions according to a multinomial process with occasion-specific entry probability *β*_*k*_,

$$\beta_{1:K}\sim Dirichlet(b_{1:K})$$ (2)

For purposes of working with an augmented data set, entry probabilities are re-expressed as conditional probabilities, *η*_*k*_, the probability of entry at *k* conditional on having not yet entered. Here,

$$\eta_{1}=\beta_{1}$$ (3)

$$\eta_{k}=\beta_{k}/(1-\sum_{l=1}^{k-1}\beta_{l}),k=2,3,\ldots K$$ (4)

The sequential state process (i.e., recruitment and survival) for individual *i* on occasion *k* (*z*_*ik*_), is now described as,

$$z_{i1}\sim Bernoulli(\eta_{1})$$ (5)

where *z*_*i*1_ = 1 if individual *i* was recruited by occasion 1 and 0 otherwise. For occasions >1, an individual may either enter if not previously entered or survive if previously present,

$$z_{ik}\sim Bernoulli(\phi z_{ik-1}+\eta_{k}\prod_{l=1}^{k-1}\left(1-z_{il}\right))$$ (6)

where *ϕ* is survival probability and the term $\prod_{l=1}^{k-1}\left(1-z_{il}\right)$ restricts entry to only include individuals that have not yet entered [25]. Combined with the latent inclusion variable (*w*_*i*_), the product *z*_*ik*_ *w*_*i*_ = 1 if individual *i* was alive and in the study population in year *k* and zero otherwise. Individuals are then detected with probability *p* conditional on being in the study population in year *k*,

$$y_{ik}\sim Bernoulli(pz_{ik}w_{i})$$ (7)

where *y*_*ik*_ denotes the detection or non-detection of individual *i* in year *k*. Annual abundance (*N*_*k*_) and the superpopulation (N*) are derived as the sum of individuals alive in year *k* and those ever alive, respectively;

$$N_{k}=\sum_{i=1}^{M}z_{ik}w_{i}$$ (8)

$$N^{*}=\sum_{i=1}^{M}w_{i}.$$ (9)

For notation simplicity, we did not include individual (*i*) or time (*k*) indices on survival and detection parameters; however, inclusion of covariates, fixed and random effects, individual heterogeneity, and multi-state formulations are common extensions of the JS model [16, 21, 34–36] and are applicable to our age-structured JS model.

### Inclusion of age structure

Age data (*x*_*ik*_; the numeric age of individual *i* in year *k*) provide information on the underlying state process (*z*_*ik*_). For example, we know that an individual first captured at occasion *k* with annual age > 0 was alive in previous sampling occasions (assuming geographic closure; see Discussion). Age data, however, are only available for observed individuals and unknown for all augmented individuals. To address this challenge, we treat age of individual *i* at occasion 1, *x*_*i*1_, as a random variable described by an initial age distribution (***π***). For indexing purposes, we assume,

$$\left(x_{i1}+1)\sim Categorical(\pi_{1:(J+1)}\right)$$ (10)

where *J* is the maximum possible age in year 1, and the first age category (*π*_1_) denotes age 0 individuals that have not yet entered. We use (*x*_*i*1_ + 1) so that age-0 (not yet entered) references the first category (*π*_1_). We define ***π*** in two parts by using the fact that *π*_1_ is equivalent to 1 − *η*_1_ in the JS model (i.e., not entered at occasion 1; Eq 3). Here, *π*_1_ = (1 − *η*_1_) is the probability that an individual has not yet entered by occasion 1 (*x*_*i*1_ = 0), and $\pi_{1:J}^{'}$ describes the initial age structure at occasion 1 conditional on being alive. Together, $\pi_{1:(J+1)}=(({1-\eta }_{1}),(\eta_{1}\pi_{1:J}^{'}))$. Parameterizing the model using an initial age structure also implies the state of an individual in year 1 is a deterministic function of age,

zi1=1ifxi1>00ifxi1=0 (11)

replacing Eq 5 in the JS model. Individuals then age annually during occasions 2, 3, … *K*,

$$x_{ik}=(x_{ik-1}+1)(1-\prod_{l=1}^{k}\left(1-z_{il}\right))$$ (12)

where annual ages are zero until an individual is recruited and increase deterministically thereafter. Missing age information for some portion of observed individuals can be addressed by treating missing age data as random variables that are estimated using the same process as the unknown ages of all augmented individuals (Eqs 10–12). Age (*x*_*ik*_) is estimated for all individuals in the population (observed and unobserved) and thus reflects the population-level age-structure, which can vary from annually observed ages for a variety of reasons (e.g., small sample sizes, variation in detection by age; see Case Study Results). Our approach makes no assumptions about stable age distributions or asymptotic properties but instead allows age structure to reflect data collected across the entirety of the study.

Directly linking the state and aging processes (Eqs 10–12) provides multiple benefits. Age data now inform the state process for all previous occasions, because recruitment year and previous survival are known (e.g., an individual first captured at age 10 is known to have been alive during the previous 9 years). Additionally, the realized age structure can be derived for any occasion as the proportion of individuals alive in each age (i.e., $N_{k}^{j}/N_{k}$, where $N_{k}^{j}$ is the number of individuals aged *j* in year *k*), providing the ability to quantify annual age structure, uncertainty in annual age structure, and investigate changes in age structure through time. Age-structured JS models also allow investigation of age-specific hypotheses such as age-specific variation in reproduction, survival, life expectancy, and density-dependence within the JS framework (analogous to models that condition on first capture; [7]). For example, we can model survival as a function of age (e.g., a quadratic function of age; [23, 37]),

$$logit(\phi_{ik})=\alpha_{0}+\alpha_{1}x_{ik}+\alpha_{2}x_{ik}^{2}$$ (13)

where *α*_0_, *α*_1_, *α*_2_ describe the survival intercept at age 0 (or some centered value) and relationships of survival with age and age^2^, respectively. Incorporating additional covariates, fixed and random effects, or individual heterogeneity on survival, recruitment, and detection parameters follow the same approaches as in JS models [16, 21, 34–36].

### Simulation study

We developed two simulation studies to evaluate model performance. We generated and analyzed 200 datasets with N* = 400 individuals, *K* = 7 occasions, occasion-specific recruitment probabilities (***β***) = (0.4, 0.1, 0.1, 0.1, 0.1, 0.1, 0.1), detection probability (*p*) = 0.25, and *J* = 9 initial age classes with an initial age distribution (***π***′) = (0.27, 0.17, 0.14, 0.12, 0.11, 0.09, 0.06, 0.03, 0.01). Settings reflect a general survey design applicable across a variety of studies, but also addresses challenges in our case study, specifically a medium duration study (7 years) with relatively low annual detection probability, an imperfectly observed age-structure, and the possibility of age-specific or age-constant survival. In the first simulation study, we assumed constant survival across ages (*ϕ* = 0.85; hereafter ‘constant-survival simulation’), while in the second simulation study we assumed survival was a quadratic function of age, $logit\left(\phi_{ik}\right)=logit\left(0.85\right)-0.5x_{ik}-0.20x_{ik}^{2}$, where ages were centered at 5 years (i.e., *x*_*ik*_ − 5) in the regression model to improve convergence (hereafter ‘age-specific survival simulation’). Although not necessary, we found that centering ages aided convergence similar to centering or scaling covariates [38]. Expected survival probabilities in the age-specific survival simulation varied from 0.63 at age 1, to a maximum of 0.88 at age 4, then decreased to 0.03 at age 9. Based on these parameter combinations, population growth rates were slightly >1.0 for the constant-survival simulations and slightly <1.0 for age-specific survival simulations. We selected nine age classes for demonstration purposes, but recognize that the number of age classes will vary by study species (e.g., studies specific to polar bears and other long-lived species will require more age classes). Although beyond the scope of this paper, simulation settings are easily modified to investigate a variety of extensions and study-specific questions including time-varying demographic rates, individual heterogeneity in detection or survival, unobservable age classes, and varying levels of missing age data (see Discussion, Supplementary Materials).

We analyzed data from the constant-survival simulation using both the JS and age-structured JS models to assess effects of including age data on the precision of parameters (*p*, ***β***, ***α***, ***π***′, *ϕ*, N*), derived annual abundances (*N*_*k*_), and annual population growth rates (*N*_*k*+1_/*N*_*k*_), which are often a primary interest in JS studies. We calculated the percent reduction in coefficient of variation for survival probabilities, annual abundances, and annual population growth rates to evaluate changes in precision between the JS and age-structured JS models for these key parameters. For the age-specific survival simulation, we used the age-structured JS model with a quadratic survival model. Here, our primary objective was to assess the ability of the age-structured JS model to return unbiased parameter estimates, particularly for age-specific survival.

We repeated analyses of both the constant and age-specific survival simulations using different values of maximum age in year 1 (*J*). The value of *J* should be, at minimum, equal to the maximum observed age in the dataset. However, deciding whether and how much larger than the maximum observed age *J* should be requires consideration of species biology and study design. To address this concern, we evaluated model robustness to selection of *J* by generating data using *J* = 9 while fitting models that assumed *J* = 9, 10, or 14 in analyses. Due to the general robustness of the model to selection of *J*, we present results from analyses assuming *J* = 10 in the main text while results from analyses assuming *J* = 9 or 14 are provided as a Supplement (S1–S3 Tables in S1 Appendix). We did not explicitly evaluate the effects of setting *J* too low as many of the simulated datasets observed at least one individual that was 8 or 9 years old during the study.

### Case study

We used a 7-year dataset (2012–2018) of individually marked polar bears in WHB to investigate multiple components of polar bear demography. The data are from a long-term study on polar bear ecology in the Hudson Bay region [28]. Our primary objective was to apply the age-structured JS model to estimate survival, recruitment, abundance, and annual age structure. The resulting estimates are from a subset of the larger, long-term dataset and do not reflect the status of the WHB polar bear subpopulation [28], but instead reflect a simplified case study to demonstrate the age-structured JS model. We analyzed encounter histories of independent polar bears (i.e., females and males age ≥ 2 years of age) monitored each fall (August—September). Each year, researchers from Environment and Climate Change Canada (ECCC) captured polar bears during helicopter surveys using standard chemical immobilization techniques [39]. Unmarked bears were individually marked by numbered ear tags and permanent tattoos. Numeric age was assigned based on analysis of a vestigial premolar extracted during first capture [28, 40]. Age was known for individuals that were first captured as cubs-of-the-year or yearlings accompanying an adult female. Capture and handling methods were reviewed and approved annually by the ECCC Western and Northern Animal Care Committee. A complete description of survey methods is provided in [28].

We fit three models to the data set: JS model without age data, age-structured JS model with constant survival, and age-structured JS model where survival was a quadratic function of age [23, 37]. We set the maximum age in year 1 at *J* = 35, which is 3 years older than the maximum observed age recorded in WHB and likely greater than the maximum age of polar bears in this region [28, 33]. For the age-specific survival model, ages were centered on the median observed age of 11 years. In addition to direct estimation of survival, recruitment, abundance, and age structure, we also derived several other metrics of ecological importance: cumulative survival, life expectancy, and annual age structure. Cumulative survival and life expectancy metrics are calculated from posterior samples of age-specific survival, while annual age structure was derived from age-specific abundances ($N_{k}^{j}/N_{k}$). Life expectancy is defined as the expected number of years that an age-2 bear (i.e., individuals that survive to independence) will survive. Life expectancy was derived as the expectation of successive binomial trials, specifically we calculate the cumulative survival probability to and death at age *j* ($\theta_{j}=\prod_{a=2}^{j-1}\phi_{a}\times \left(1-\phi_{j}\right)$), then sum expectations across all ages ($life\;expectancy=\sum_{a=2}^{J}{a\theta }_{a}$). Additionally, we monitored the annual proportion of the population in prime breeding age (10–15 years old), a period when polar bears often exhibit their greatest reproductive output [32, 41]. We assumed constant detection probability (*p*) across years, conditional on an individual being alive (Eq 7).

### Implementation

Models were fit in a Bayesian framework using Markov chain Monte Carlo (MCMC) methods. Both the JS and age-structured JS models are easily fit in common MCMC software packages such as WinBUGS, JAGS, or NIMBLE. In our study, models were fit using NIMBLE v0.8.0 [42] accessed through R v3.5.1 [43]. For the simulation studies, we ran three chains for 120,000 iterations with 20,000 iterations discarded as burn-in and thinned to every 10^th^ iteration to reduce file size. For the case study, we increased the number of chains to six and the number of iterations to 220,000 to increase the number of effective samples. We assessed convergence using diagnostic plots and the Gelman-Rubin statistic ($\hat{R}$; [44]). Vague priors were used for all parameters [44, 45], specifically Beta(1,1) for detection probability (*p*) and the data augmentation parameter (*ψ*), and separate Dirichlet(**1**) priors for entrance probabilities (***β***) and initial age distribution (***π***′). For the constant-survival models, we used *ϕ*~Beta(1,1), and for the quadratic survival models we assumed inv.logit(*α*_0_) ~Beta(1,1) and independent Normal(0, sd = 10) for *α*_1_ and *α*_2_. Results are reported as posterior medians and 2.5 and 97.5 percentiles (95% CRI) of retained posterior samples.

We evaluated goodness-of-fit using a posterior-predictive check to evaluate the ability of the model to predict the number of observed individuals each year (*n*_*k*_), which is a shared metric across modelling approaches. For each iteration, we generated the expected number of observed individuals (${\tilde{n}}_{k}\sim Binomial(p,N_{k})$) and compared the observed and expected counts using the Freeman-Tukey statistic [34, 46]. There was no evidence of a lack of fit for any of the models (Bayesian p-values = 0.39 for JS, 0.43 for the age-structured JS with constant survival, and 0.59 for age-structured JS with age-specific survival). R scripts and model code for the simulation and case studies are provided as Supplementary Materials (S1–S3 Files). Under our simulation settings, each model generally required < 5 hours to run on a desktop with a 3.1 GHz processor.

## Results

### Simulation study

**Fig 1:**
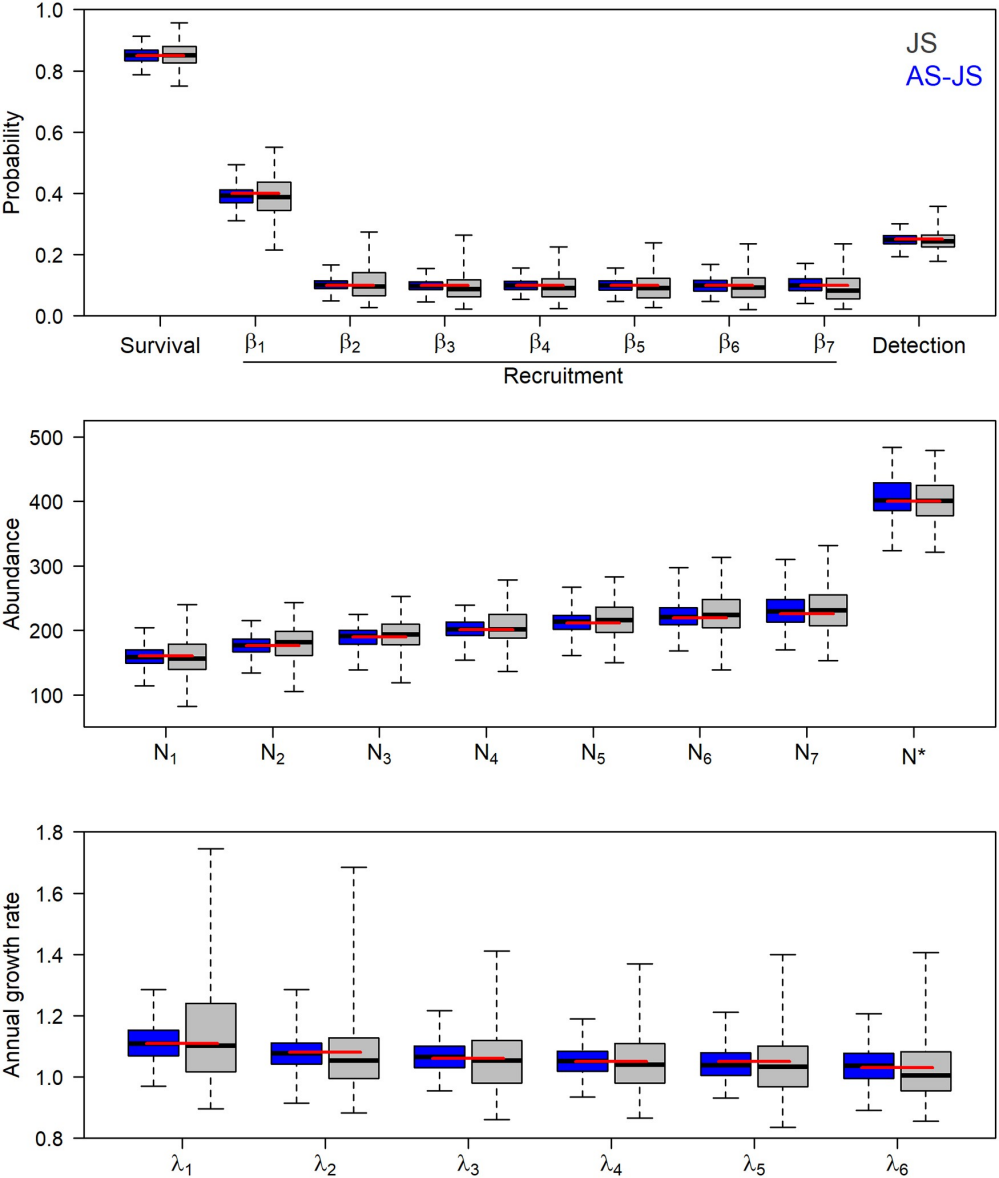
Boxplots of posterior medians for survival, recruitment, and detection (top), abundances (middle), and annual population growth rates (bottom) from 200 simulated data sets analyzed using Jolly-Seber models that ignore (grey) or incorporate (blue) age structure. Boxplots include medians (black line), interquartile range (box), and range of values (whiskers). Red horizontal lines denote data generating values. Data generation fixed the maximum age in year 1 (*J*) at 9, but age structure analyses assumed *J* = 10 to evaluate robustness to uncertainty in maximum age.

Both the JS and age-structured JS model produced unbiased estimates of survival, recruitment, and abundance in the constant-survival simulations (Fig 1, S1 Table in S1 Appendix). The age-structured JS model improved precision of all parameters relative to the JS model without age data (Fig 1). Incorporation of age structure also reduced fluctuations between successive *N*_*k*_, resulting in improved precision of annual growth rates (Fig 1). Under these simulation settings, the coefficients of variation for survival, annual abundance, and annual population growth rate were reduced by 32%, 35%, and 52%, respectively, in the age-structured JS model relative to the JS model (Fig 1).

**Fig 2:**
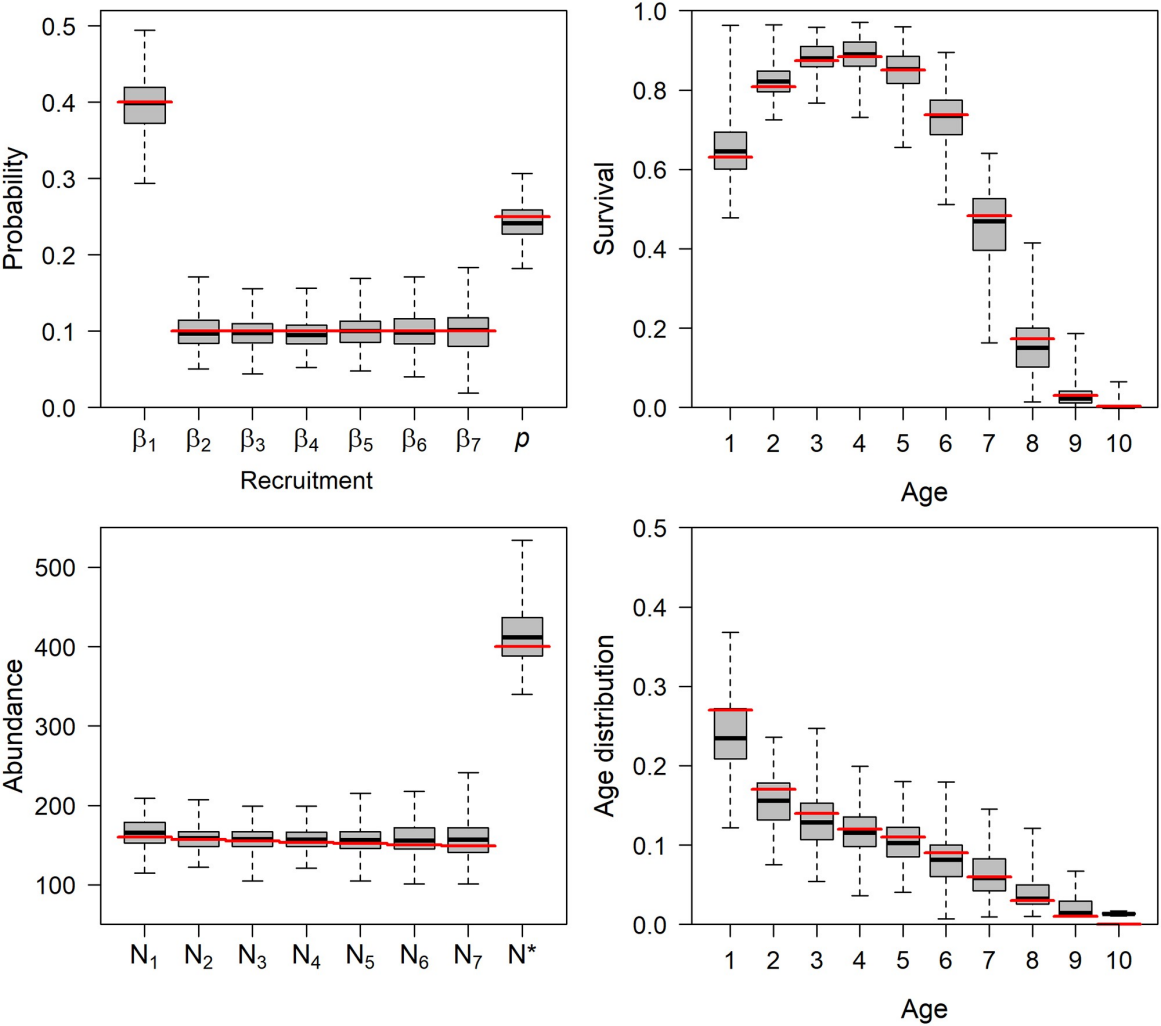
Boxplots of posterior medians for annual recruitment and detection (*β*_*k*_ and *p*, respectively; top left), age-specific survival (top right), abundances (bottom left), and initial age distribution (bottom right) from 200 simulated data sets analyzed using age-structured Jolly-Seber models where survival is a quadratic function of age. Boxplots include medians (black line), interquartile range (box), and range of values (whiskers). Red horizontal lines denote data generating values. Data generation fixed the maximum age in year 1 (*J*) at 9, but analyses assumed *J* = 10 to evaluate robustness to uncertainty in maximum age.

The age-structured JS model also performed well in age-specific survival simulations, providing minimally biased to unbiased estimates of age-specific survival, abundance, recruitment, and initial age structure (Fig 2). A slight negative bias was observed in the initial age distribution for age 1 individuals. This likely resulted from several factors, including small sample size bias as numerous simulations resulted in zero individuals aged 8- or 9-years-old in year 1, low detection probability, and simulation of true maximum age *J* = 9 but with the assumption during analysis of *J* = 10. To evaluate the effect of detection probability (*p*) on the estimation of age structure, we re-ran simulations where *p* = 0.50 and bias noticeably decreased (S3 Table in S1 Appendix). Survival, recruitment, detection, and most abundance estimates appeared robust to the selection of *J* (S1–S3 Tables in S1 Appendix). Biases in year 1 abundance, superpopulation abundance, and year 1 age structure were evident when modeled *J* ≫ true *J* (i.e., true *J* = 9 but was modeled as 14), but diminished with increasing detection probability and increasingly reasonable selection of *J* (i.e., *J* = 9 or 10; S1–S3 Tables in S1 Appendix).

### Case study

**Fig 3:**
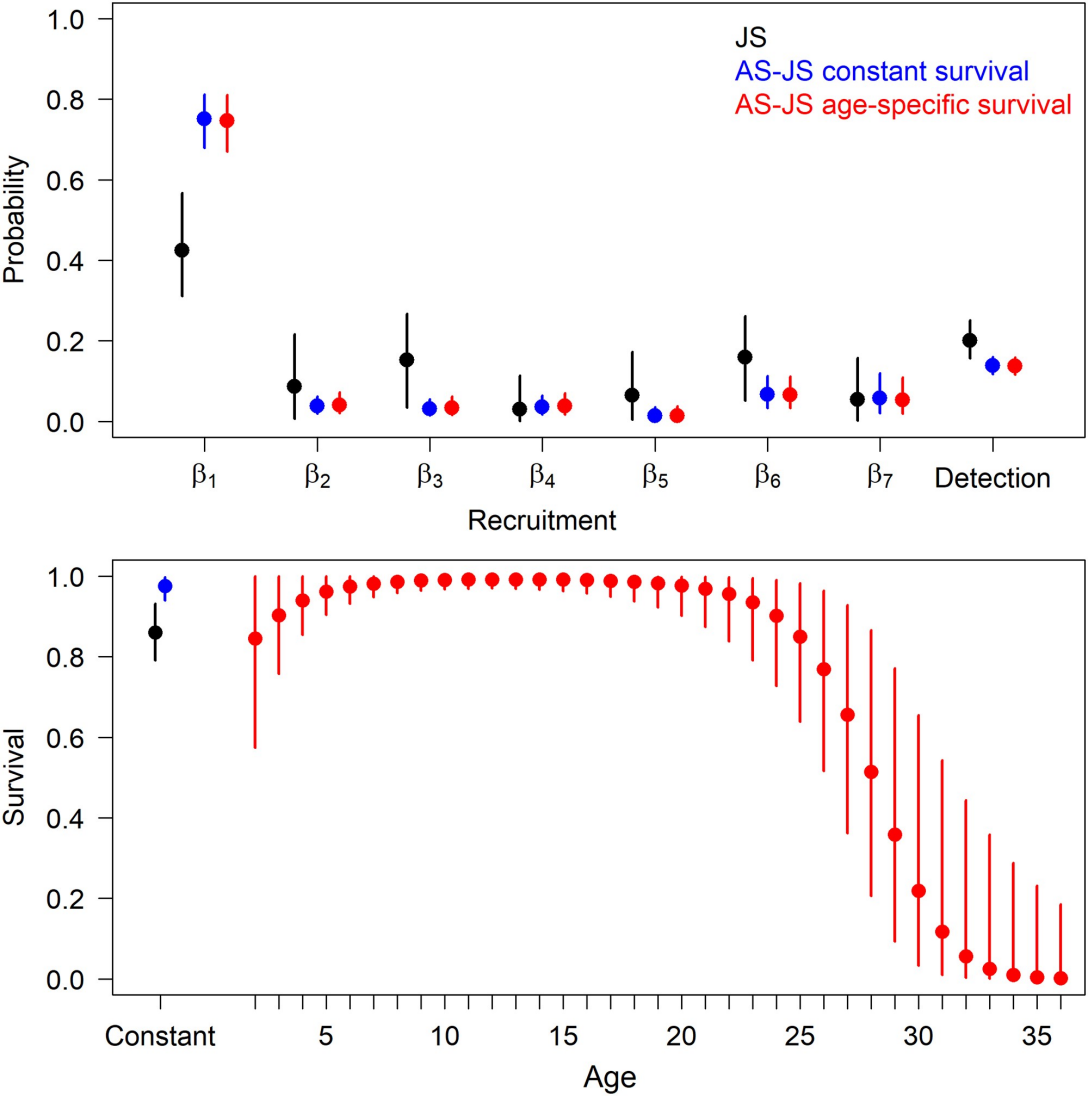
Recruitment and detection probabilities (top) and annual survival (bottom) of western Hudson Bay polar bears from Jolly-Seber models that ignore age structure (black) or incorporate age structure and assume annual survival is constant (blue) or a quadratic function of age (red). Points and error bars are posterior medians and 95% credible intervals, respectively.

We analyzed encounter histories from *n* = 296 individual polar bears that included 427 capture events. The number of bears captured each year ranged from 51 to 72 independent bears, with observed ages from 2–30 years old. Abundance estimates from the age-structured JS model with constant and quadratic survival functions were relatively consistent but differed from the JS model without age data. Most bears in the superpopulation were present at occasion 1 in the age-structured JS models (Fig 3). Survival in the age-structured JS model with constant survival was 0.98 (95% CRI: 0.94–1.00), substantially higher than the JS model without age data (0.86, 95% CRI: 0.79–0.93; Fig 3). The age-structured JS model with quadratic survival supported the hypothesis of survival senescence, with survival lower for younger individuals, near >0.95 for individuals aged 7–22, then decreasing to near zero for individuals > 30 years (Fig 3).

**Fig 4:**
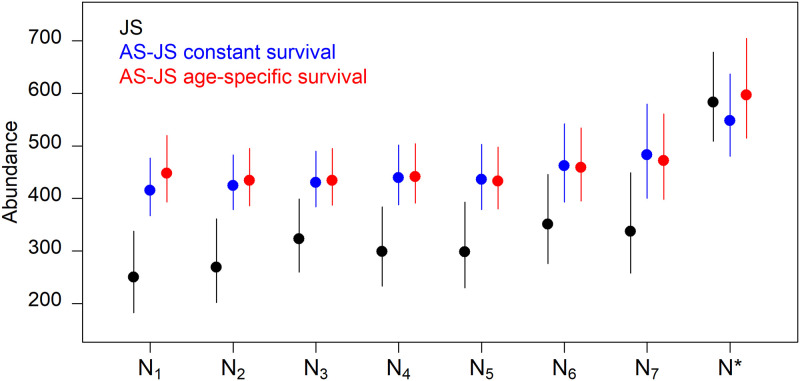
Annual and superpopulation abundances for western Hudson Bay (WHB) polar bears from Jolly-Seber models that ignore age structure (black) or incorporate age structure and assume annual survival is constant (blue) or a quadratic function of age (red). Points and error bars are posterior medians and 95% credible intervals, respectively. Analyses used a subset of the larger, long-term WHB polar bear study and, therefore, do not reflect the status of the entire subpopulation ([28]; see Methods for additional details).

**Fig 5:**
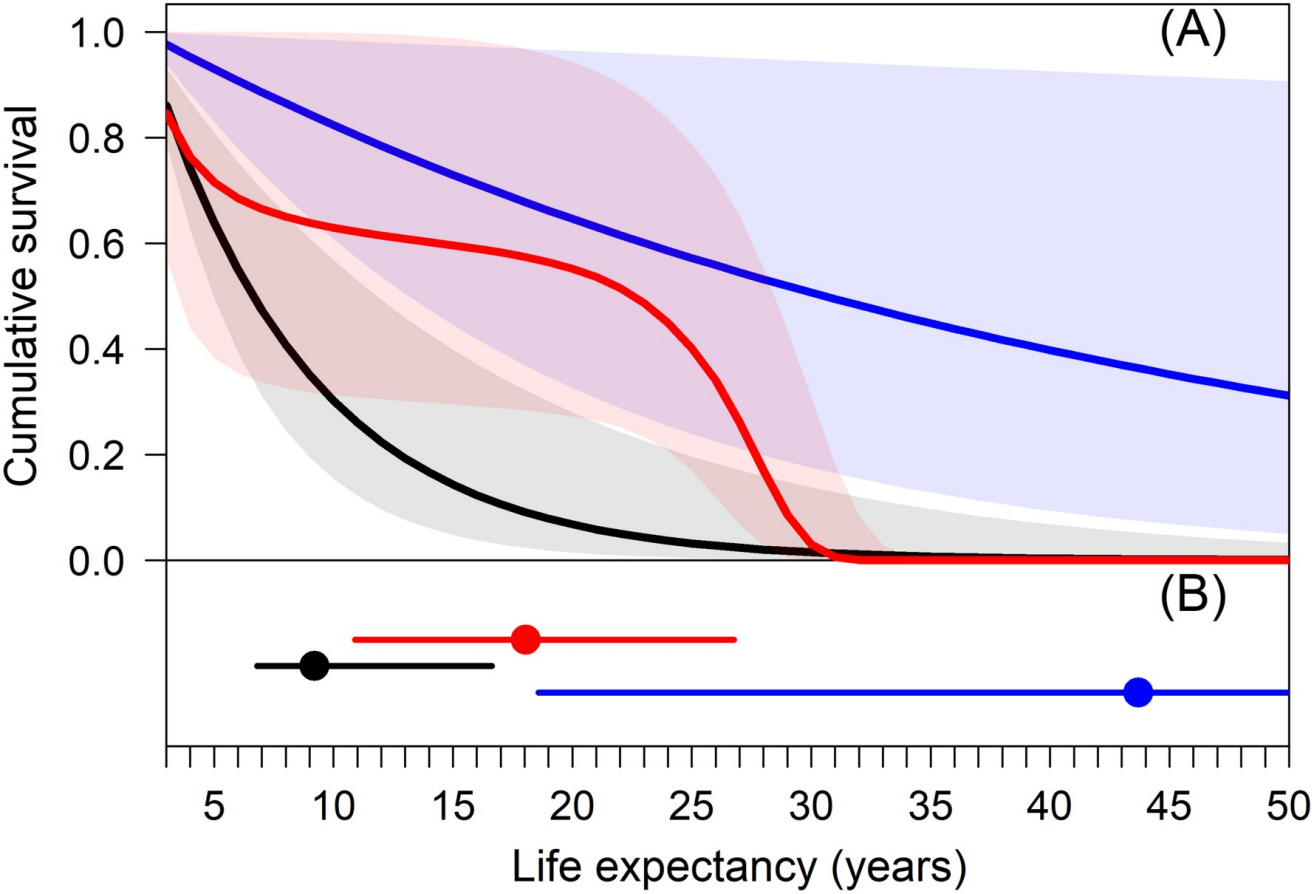
Cumulative survival (A) and life expectancy (B) of western Hudson Bay polar bears from Jolly-Seber models that ignore age structure (black) or incorporate age structure and assume annual survival is constant (blue) or a quadratic function of age (red). Posterior medians and 95% credible intervals are shown. Cumulative survival and life expectancy estimates are conditional on surviving to two years of age (i.e., independent bears). Upper credible bound for life expectancy from the age structure model with constant survival is > 100 years and not shown (see Results).

All models provided similar estimates of superpopulation abundance (~ 500–600 individuals) but differed in the survival and recruitment processes leading to these superpopulation abundances (Fig 4). Ignoring age structure resulted in lower survival, lower year 1 abundance, and higher and more variable recruitment in subsequent years, leading to substantial variation among yearly abundance estimates (Figs 3 and 4). Conversely, in the age-structured JS model, abundance estimates were higher, more precise, and relatively consistent across years due to higher annual survival and lower recruitment, leading to more biologically plausible values for a K-selected species (Figs 4 and 5). Conditional on surviving to independence (age 2), life expectancies from the JS model and the age-structured JS model with quadratic survival were 9.2 yrs (6.8–16.6) and 18.1 yrs (10.9–26.8), respectively. Life expectancy from the age-structured JS model with constant survival was 43.7 yrs (18.6–100+ yrs) with the upper credible interval never stabilizing due to posterior mass of annual survival near 1.0 (Fig 5). While polar bears are long lived, individuals > 25 years-old are rarely observed [28, 33]. Life expectancy results provide further ecological support for the age-structured JS model with quadratic survival while demonstrating how small changes in annual survival lead to large differences in life expectancy for long-lived species (Fig 5).

**Fig 6:**
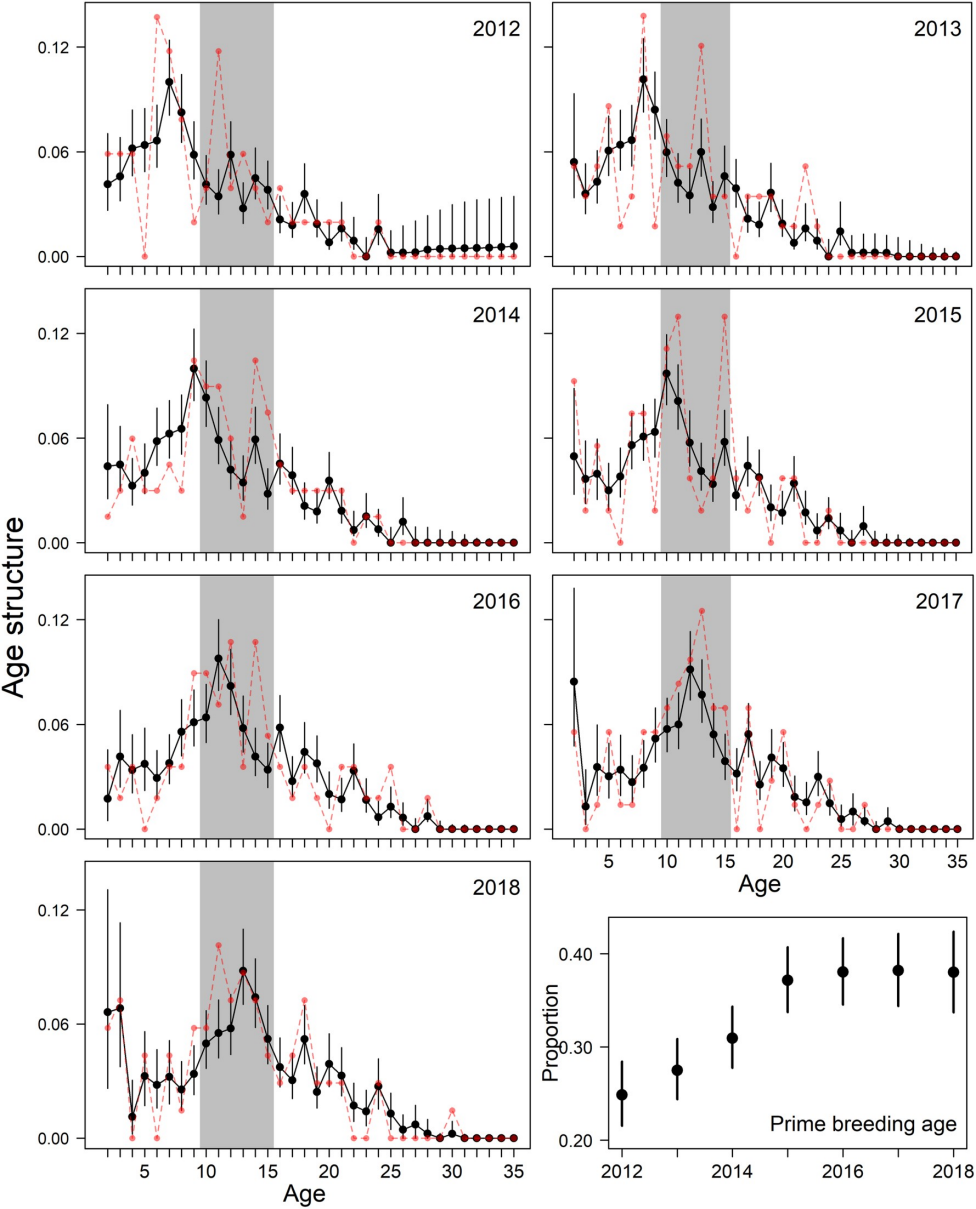
Annual age structure of western Hudson Bay polar bears using an age-structured Jolly-Seber model that assumes annual survival is a quadratic function of age (black; medians and 95% credible intervals). Red points are year-specific proportions from observed data. Grey polygon denotes prime breeding ages (10–15 years of age). The annual proportion of the population in prime breeding age is summarized in lower right panel (median and 95% credible intervals).

Individuals 6–8 years of age were disproportionately represented in the 2012 age structure, suggesting strong recruitment from the 2004–2006 birth years. Cascading effects of this large cohort led to significant changes in population age structure and recruitment across years (Fig 6). The proportion of individuals in prime breeding age (10–15 years of age) varied among years, increasing from 0.25 in 2012 (95% CRI: 0.22–0.28) to 0.38 in 2016–2018 (95% CRI: 0.34–0.42), which tracked the aging of the 2004–2006 cohort (Fig 6). Recruitment of age 2 individuals was lower during 2012–2016 but increased in 2017 and 2018, 2 years after the large cohort born in 2004–2006 entered prime breeding ages (Fig 6). Explicitly linking survival, recruitment, and aging processes also forced ecologically consistent changes in age structure across years (Fig 6). For example, although surveys detected zero age 5 individuals in 2012, the estimated proportion of age 5 individuals was ≫ 0 due to observations of this age class in subsequent years (Fig 6). Similarly, changes in the estimated annual age structure followed biologically plausible processes even though annual age data displayed large fluctuations due to small annual sample sizes (Fig 6).

## Discussion

We developed an age-structured JS model to improve estimation of demographic parameters and, thus, inference about population dynamics from capture-recapture and age data. The novelty of our approach arises from integrating model components describing age structure, aging, survival, recruitment, and abundance into a single hierarchical model that overcomes the challenges of unknown ages in JS models (for observed and unobserved individuals) and individuals born prior to the study [15]. Unifying these processes within the JS framework provides a holistic approach to evaluating the effects of age structure on population dynamics, while substantially improving precision of demographic parameter estimates. The additional information that comes from our age-structured JS model has numerous applications in population and evolutionary ecology, including identification of sustainable harvest levels that reflect age-related variation in reproductive value [47], survival or reproductive senescence [1], and understanding the influence of age structure on population viability [2, 48].

Evaluating age-specific demography, particularly demographic senescence (i.e., degradation of survival or breeding probabilities associated with aging), is essential to understanding the impacts of age structure on population dynamics [1]. If demographic senescence is ignored, parameters such as life expectancy, extinction risk, and population viability may be over- or underestimated [2]. We found that WHB polar bear survival increased with age early in life and reached a plateau from approximately 7–22 years of age, followed by decreasing survival thereafter, a pattern common in many mammal species [49]. Survival of polar bears previously defined as “prime-age” (i.e., 5–19 years of age; *sensu* [30]) was high and relatively stable, which has been hypothesized as an effect of improved body condition, enhanced hunting efficiency, and intra-species interactions during these ages [28–30]. Polar bear reproductive senescence was previously documented and primarily attributed to changes in body condition [32, 50, 51]; however, survival senescence is less well understood but may have important implications for the viability of K-selected species such as polar bears [1, 30, 31].

Individuals 6–8 years of age were disproportionately represented in the 2012 age structure, suggesting large recruitment classes in 2004–2006. WHB female body mass was above average when these large age classes were dependent young (2005–2009; Lunn unpublished data), supporting previous findings that maternal body mass in polar bears is positively correlated with increased reproduction and survival of their young [50, 51]. Increased recruitment of age 2 individuals during 2017–2018 coincides with years when the 2004–2008 cohort began entering prime breeding ages (10–15 years old; Fig 6; [28, 32, 51]), providing a possible mechanism to explain this increase in recruitment. Results thus far demonstrate how unifying age structure and demographic models can provide new insights into the effects of age structure on population dynamics, growth, and viability, but further investigations are required to separate variation in recruitment due to environmental conditions, increased abundance of breeding age individuals, and favorable conditions early in a breeder’s life [32].

The generality of our approach provides a promising tool for future investigations into the effects of aging on population dynamics. Thus far, we have assumed age can be identified when an individual is observed. Although not fully explored herein, our approach allows for both missing age data and the incorporation of age proxies (e.g., morphometric data such as dental cementum or size) when annual age data are missing. Missing age data for some portion of observed individuals are addressed using the same process as unknown ages for augmented individuals, whereby age in year one is considered a random variable (Eqs 10–12). As the proportion of observed individuals without age data increases, information on the age-structure declines and the age-structured JS model reverts to a JS model without age data. The levels at which missing age data result in no additional benefits, however, will likely vary by study species (e.g., maximum age and life expectancy) and survey design (e.g., detection probability, study duration). In the absence of explicit age data, age proxies could be integrated with an additional hierarchical level that describes, for example, a growth model linking morphometric data to annual age (e.g., Gompertz, von Bertalanffy) [5, 11]. Growth model parameters can be directly estimated when both morphometric data and age data are available for some individuals. Alternatively, informative priors can be developed from separate studies linking morphometric data to age [52].

Simulation studies demonstrated that both the JS and age-structured JS models provided unbiased estimates of demographic rates and abundances; however, incorporating age data improved precision of demographic rates and population growth rates, increased the power to detect trends in abundance, and allowed unbiased estimation of age-dependent survival and changes in annual age structure. The age-structured JS model was generally robust to uncertainty in the selection of maximum age in year 1 (*J*), with parameter estimates practically unaffected even when *J* was 1.5 times greater than the true value. Setting *J* requires thoughtful consideration of species biology; however, setting *J* too high will often be apparent via estimates of age structures ≤ 0.01 across consecutive older age classes (S1 Appendix). Finally, while our simulation studies were not exhaustive, the simulation scripts provided in S1 File are readily modified and provide opportunities to extend this general framework to investigate study-specific topics. As noted by reviewers, there are numerous extensions worthy of future research, including the incorporation of covariates on demographic rates, individual heterogeneity in detection or survival, unobservable age classes, and the effects of missing age data [16, 21, 34–36, 53].

We made several simplifying assumptions in our case study by not allowing for individual or temporal variation in survival (except by age), detection, reproduction, or movement, although there is capacity within our framework to generalize the model to these factors. Our case study consisted of a subset of a broader, long-term WHB polar bear study, thus our results may not represent the status of the entire subpopulation and are not intended to be used for management purposes [28]. Also, the age-structured JS model described herein is non-spatial and assumes individuals recruit into the population when born or at independence (e.g., 2 years of age in our polar bear case study). In this parameterization, recruitment provides direct insights into intrinsic recruitment factors (i.e., reproduction) but does not explicitly handle spatial processes such as immigration. High proportions of older immigrants could result in positive bias in annual abundance and survival as older immigrants are assumed to be alive and in the population during previous occasions. Extending our non-spatial age-structured JS to a spatially explicit open population model [54, 55] may help distinguish between demographic (recruitment, aging, and survival) and geographic (immigration and emigration) processes, while providing more robust ecological inferences from combined capture-recapture and age data.

Age structure of free-ranging populations may fluctuate in response to environmental stressors, especially factors that disproportionately affect reproduction or age-dependent survival (e.g., weather, competition; [5, 56]). For long-lived species such as polar bears, birth rate and survival probability of newly independent animals (i.e., age 2 years) often respond first to regulating factors such as declining carrying capacity [47, 57]. Shifts in age structure can affect population dynamics, resulting in population growth rates and viability measures that vary considerably from asymptotic projections [3, 6, 58]. The degree to which age structure varies in free-ranging populations, however, is poorly understood, because the data required to estimate annual age structure have been difficult to obtain [3, 6]. Conversely, we demonstrate how an age-structured JS model provides a flexible approach to jointly estimate population-level annual age structure, abundance, and demographic rates from commonly collected capture-recapture and age data. For example, our case study detected a substantial pulse in recruitment associated with an increase in the proportion of prime breeding age adults even though the model did not explicitly force this relationship. We believe this age-structured JS model provides numerous opportunities to explore age structure dynamics and how these dynamics result in fluctuations in vital rates and the trajectories of free-ranging populations.

Jointly modeling abundance, survival, recruitment, age structure, and the aging process within the JS framework provides an important advance in our ability to evaluate population dynamics and provides crucial information for species management and conservation. Integration of age and capture-recapture data within the JS framework allows exploration of a wider range of demographic processes, including evolutionary and life history analyses (e.g., senescence, life expectancy, reproductive success) and the effects of age structure on population persistence, while also improving our ability to explore interacting hypotheses in evolutionary, behavioral, and population ecology. Recognizing how demographic rates, abundance, and age structure interact within the JS framework in turn can help improve the explanatory power of JS models and more accurately forecast future population dynamics.

## Supporting information

S1 Appendix S1–S3 Tables summarizing simulation results. (DOCX)

S1 File R script to generate and analyze age-structured mark-recapture data described in the manuscript. (R)

S2 File Data associated with the case study. (TXT)

S3 File R script to load, format, and run the models described in the case study. (R)
