# Correction: Age-structured Jolly-Seber model expands inference and improves parameter estimation from capture-recapture data

**DOI:** 10.1371/journal.pone.0271784

**Published:** 2022-07-14

**Authors:** PLOS ONE

There is an error in affiliation 2 for author Nicholas J. Lunn. The correct affiliation is: Wildlife Research Division, Science and Technology Branch, Environment and Climate Change Canada, Edmonton, Alberta, Canada.

The affiliation for the third author is also incorrect. The correct affiliation is not indicated. Evan S. Richardson is not affiliated with #2 but with: Wildlife Research Division, Science and Technology Branch, Environment and Climate Change Canada, Winnipeg, Manitoba, Canada.

The publisher apologizes for the errors.
