# Supplementary material for: Age-structured Jolly-Seber model expands inference and improves parameter estimation from capture-recapture data
Source: PLoS One. 2021 Jun 9;16(6):e0252748. doi: 10.1371/journal.pone.0252748 (PMC8189494; doi:10.1371/journal.pone.0252748)
Supplement: S1 Appendix — (DOCX) [file pone.0252748.s001.docx]

**SUPPORTING INFORMATION**

Hostetter NJ, NJ Lunn, ES Richardson, EV Regehr, and SJ Converse. Age-structured Jolly-Seber model expands inference and improves parameter estimation from capture-recapture data.

**Appendix S1**. Supplementary Tables.

**Authors**

Nathan J. Hostetter^1*^, Nicholas J. Lunn^2^, Evan S. Richardson^2^, Eric V. Regehr^3^, Sarah J. Converse^4^

^1^ Washington Cooperative Fish and Wildlife Research Unit, School of Aquatic and Fishery Sciences, University of Washington

^2^ Wildlife Research Division, Science and Technology Branch, Environment and Climate Change Canada

^3^ Polar Science Center, Applied Physics Laboratory, University of Washington

^4^ U.S. Geological Survey, Washington Cooperative Fish and Wildlife Research Unit, School of Environmental and Forest Sciences (SEFS) & School of Aquatic and Fishery Sciences (SAFS), University of Washington

Table S1. Detailed simulation results comparing expected values (mean), root mean squared error (RMSE), and credible coverage (Cov) from 200 simulated data sets analyzed using Jolly-Seber models that ignore age structure (Jolly-Seber) or integrate age structure (AS-JS). Data simulations set maximum age in year 1 ($J$) as 9, and we fit age-structured Jolly-Seber models assuming $J$ = 9, 10, or 14. See methods for complete parameter definitions and simulation settings.

|  |  | Jolly-Seber | | |  | AS-JS (J = 9) | | |  | AS-JS (J = 10) | | |  | AS-JS (J = 14) | | |
| --- | --- | --- | --- | --- | --- | --- | --- | --- | --- | --- | --- | --- | --- | --- | --- | --- |
| Parameter | TRUE | Mean | RMSE | Cov |  | Mean | RMSE | Cov |  | Mean | RMSE | Cov |  | Mean | RMSE | Cov |
| $N^{*}$ | 400 | 401 | 31.6 | 0.95 |  | 405 | 30.8 | 0.94 |  | 405 | 30.8 | 0.94 |  | 405 | 30.8 | 0.95 |
| $\phi$ | 0.85 | 0.85 | 0.04 | 0.96 |  | 0.85 | 0.02 | 0.96 |  | 0.85 | 0.02 | 0.96 |  | 0.85 | 0.02 | 0.94 |
| $p$ | 0.25 | 0.25 | 0.03 | 0.94 |  | 0.25 | 0.25 | 0.94 |  | 0.25 | 0.25 | 0.94 |  | 0.25 | 0.25 | 0.93 |
| $\beta_{1}$ | 0.40 | 0.39 | 0.06 | 0.96 |  | 0.39 | 0.03 | 0.92 |  | 0.39 | 0.03 | 0.92 |  | 0.39 | 0.03 | 0.91 |
| $\beta_{2}$ | 0.10 | 0.11 | 0.05 | 0.98 |  | 0.10 | 0.02 | 0.95 |  | 0.10 | 0.02 | 0.96 |  | 0.10 | 0.02 | 0.96 |
| $\beta_{3}$ | 0.10 | 0.09 | 0.04 | 0.99 |  | 0.10 | 0.02 | 0.91 |  | 0.10 | 0.02 | 0.92 |  | 0.10 | 0.02 | 0.91 |
| $\beta_{4}$ | 0.10 | 0.10 | 0.04 | 0.99 |  | 0.10 | 0.02 | 0.96 |  | 0.10 | 0.02 | 0.96 |  | 0.10 | 0.02 | 0.95 |
| $\beta_{5}$ | 0.10 | 0.10 | 0.04 | 0.98 |  | 0.10 | 0.02 | 0.96 |  | 0.10 | 0.02 | 0.96 |  | 0.10 | 0.02 | 0.95 |
| $\beta_{6}$ | 0.10 | 0.10 | 0.05 | 0.98 |  | 0.10 | 0.02 | 0.94 |  | 0.10 | 0.02 | 0.94 |  | 0.10 | 0.02 | 0.94 |
| $\beta_{7}$ | 0.10 | 0.09 | 0.05 | 0.97 |  | 0.10 | 0.03 | 0.97 |  | 0.10 | 0.03 | 0.97 |  | 0.10 | 0.03 | 0.97 |
| $\pi_{1}^{'}$ | 0.27 | - | - | - |  | 0.26 | 0.04 | 0.96 |  | 0.25 | 0.04 | 0.96 |  | 0.25 | 0.05 | 0.93 |
| $\pi_{2}^{'}$ | 0.17 | - | - | - |  | 0.16 | 0.03 | 0.96 |  | 0.16 | 0.03 | 0.96 |  | 0.16 | 0.03 | 0.92 |
| $\pi_{3}^{'}$ | 0.14 | - | - | - |  | 0.14 | 0.03 | 0.93 |  | 0.13 | 0.03 | 0.93 |  | 0.13 | 0.03 | 0.92 |
| $\pi_{4}^{'}$ | 0.12 | - | - | - |  | 0.12 | 0.03 | 0.95 |  | 0.12 | 0.03 | 0.94 |  | 0.12 | 0.03 | 0.92 |
| $\pi_{5}^{'}$ | 0.11 | - | - | - |  | 0.11 | 0.03 | 0.96 |  | 0.10 | 0.03 | 0.96 |  | 0.10 | 0.03 | 0.94 |
| $\pi_{6}^{'}$ | 0.09 | - | - | - |  | 0.09 | 0.03 | 0.94 |  | 0.09 | 0.03 | 0.95 |  | 0.08 | 0.02 | 0.95 |
| $\pi_{7}^{'}$ | 0.06 | - | - | - |  | 0.06 | 0.02 | 0.96 |  | 0.06 | 0.02 | 0.95 |  | 0.06 | 0.02 | 0.94 |
| $\pi_{8}^{'}$ | 0.03 | - | - | - |  | 0.03 | 0.02 | 0.96 |  | 0.03 | 0.02 | 0.96 |  | 0.03 | 0.02 | 0.96 |
| $\pi_{9}^{'}$ | 0.01 | - | - | - |  | 0.01 | 0.01 | 0.99 |  | 0.01 | 0.01 | 0.99 |  | 0.01 | 0.01 | 0.99 |
| $\pi_{10}^{'}$ | 0.00 | - | - | - |  | - | - | - |  | 0.01 | 0.01 | 0.00 |  | 0.01 | 0.01 | 0.00 |
| $\pi_{11}^{'}$ | 0.00 | - | - | - |  | - | - | - |  | - | - | - |  | 0.01 | 0.01 | 0.00 |
| $\pi_{12}^{'}$ | 0.00 | - | - | - |  | - | - | - |  | - | - | - |  | 0.01 | 0.01 | 0.00 |
| $\pi_{13}^{'}$ | 0.00 | - | - | - |  | - | - | - |  | - | - | - |  | 0.01 | 0.01 | 0.00 |
| $\pi_{14}^{'}$ | 0.00 | - | - | - |  | - | - | - |  | - | - | - |  | 0.01 | 0.01 | 0.00 |
|  |  |  |  |  |  |  |  |  |  |  |  |  |  |  |  |  |
|  |  |  |  |  |  |  |  |  |  |  |  |  |  |  |  |  |
| DERIVED |  |  |  |  |  |  |  |  |  |  |  |  |  |  |  |  |
| $N_{1}$ | 160 | 159 | 26.6 | 0.96 |  | 159 | 12.4 | 0.95 |  | 159 | 12.4 | 0.94 |  | 159 | 12.4 | 0.95 |
| $N_{2}$ | 176 | 181 | 23.1 | 0.98 |  | 177 | 11.6 | 0.97 |  | 177 | 11.6 | 0.97 |  | 177 | 11.6 | 0.97 |
| $N_{3}$ | 190 | 193 | 23.0 | 0.96 |  | 190 | 12.4 | 0.94 |  | 190 | 12.4 | 0.94 |  | 190 | 12.5 | 0.93 |
| $N_{4}$ | 201 | 205 | 26.4 | 0.94 |  | 202 | 13.8 | 0.96 |  | 202 | 13.8 | 0.96 |  | 202 | 13.8 | 0.95 |
| $N_{5}$ | 211 | 216 | 26.7 | 0.95 |  | 213 | 16.3 | 0.94 |  | 213 | 16.3 | 0.94 |  | 213 | 16.3 | 0.94 |
| $N_{6}$ | 219 | 226 | 30.8 | 0.94 |  | 222 | 20.3 | 0.92 |  | 222 | 20.4 | 0.92 |  | 222 | 20.4 | 0.92 |
| $N_{7}$ | 226 | 232 | 34.3 | 0.93 |  | 231 | 26.9 | 0.92 |  | 231 | 27.0 | 0.92 |  | 231 | 27.0 | 0.92 |

Table S2. Detailed simulation results comparing expected values (mean), root mean squared error (RMSE), and credible coverage (Coverage) from 200 simulated data sets analyzed using age-structured Jolly-Seber models (AS-JS) where survival is a quadratic function of age. Data simulations set maximum age in year 1 ($J$) as 9, and we fit age-structured Jolly-Seber models assuming $J$ = 9, 10, or 14. See methods for complete parameter definitions and simulation settings.

|  |  | AS-JS ($J$ = 9) | | |  | AS-JS ($J$ = 10) | | |  | AS-JS ($J$ = 14) | | |
| --- | --- | --- | --- | --- | --- | --- | --- | --- | --- | --- | --- | --- |
| Parameter | TRUE | Mean | RMSE | Coverage |  | Mean | RMSE | Coverage |  | Mean | RMSE | Coverage |
| $N^{*}$ | 400 | 412.99 | 39.68 | 0.96 |  | 415.29 | 40.57 | 0.94 |  | 423.74 | 44.98 | 0.93 |
| $\alpha_{0}$ | 1.73 | 1.77 | 0.39 | 0.96 |  | 1.79 | 0.40 | 0.97 |  | 1.83 | 0.42 | 0.95 |
| $\alpha_{1}$ | -0.50 | -0.56 | 0.15 | 0.92 |  | -0.57 | 0.15 | 0.94 |  | -0.57 | 0.15 | 0.92 |
| $\alpha_{2}$ | -0.20 | -0.21 | 0.06 | 0.98 |  | -0.21 | 0.06 | 0.98 |  | -0.22 | 0.06 | 0.97 |
| $p$ | 0.25 | 0.24 | 0.02 | 0.95 |  | 0.24 | 0.02 | 0.94 |  | 0.24 | 0.03 | 0.93 |
| $\beta_{1}$ | 0.40 | 0.40 | 0.04 | 0.94 |  | 0.40 | 0.04 | 0.94 |  | 0.41 | 0.04 | 0.95 |
| $\beta_{2}$ | 0.10 | 0.10 | 0.02 | 0.92 |  | 0.10 | 0.02 | 0.93 |  | 0.10 | 0.02 | 0.93 |
| $\beta_{3}$ | 0.10 | 0.10 | 0.02 | 0.94 |  | 0.10 | 0.02 | 0.92 |  | 0.10 | 0.02 | 0.92 |
| $\beta_{4}$ | 0.10 | 0.10 | 0.02 | 0.96 |  | 0.10 | 0.02 | 0.96 |  | 0.10 | 0.02 | 0.95 |
| $\beta_{5}$ | 0.10 | 0.10 | 0.02 | 0.96 |  | 0.10 | 0.02 | 0.96 |  | 0.10 | 0.02 | 0.96 |
| $\beta_{6}$ | 0.10 | 0.10 | 0.02 | 0.94 |  | 0.10 | 0.02 | 0.94 |  | 0.10 | 0.02 | 0.94 |
| $\beta_{7}$ | 0.10 | 0.10 | 0.03 | 0.96 |  | 0.10 | 0.03 | 0.96 |  | 0.10 | 0.03 | 0.97 |
| $\pi_{1}^{'}$ | 0.27 | 0.24 | 0.05 | 0.90 |  | 0.24 | 0.05 | 0.88 |  | 0.22 | 0.06 | 0.81 |
| $\pi_{2}^{'}$ | 0.17 | 0.16 | 0.03 | 0.95 |  | 0.15 | 0.04 | 0.94 |  | 0.14 | 0.04 | 0.89 |
| $\pi_{3}^{'}$ | 0.14 | 0.13 | 0.03 | 0.94 |  | 0.13 | 0.03 | 0.94 |  | 0.12 | 0.04 | 0.92 |
| $\pi_{4}^{'}$ | 0.12 | 0.12 | 0.03 | 0.98 |  | 0.12 | 0.03 | 0.98 |  | 0.11 | 0.03 | 0.96 |
| $\pi_{5}^{'}$ | 0.11 | 0.11 | 0.03 | 0.96 |  | 0.10 | 0.03 | 0.95 |  | 0.10 | 0.03 | 0.93 |
| $\pi_{6}^{'}$ | 0.09 | 0.08 | 0.03 | 0.93 |  | 0.08 | 0.03 | 0.92 |  | 0.08 | 0.03 | 0.92 |
| $\pi_{7}^{'}$ | 0.06 | 0.06 | 0.03 | 0.96 |  | 0.06 | 0.03 | 0.97 |  | 0.06 | 0.03 | 0.97 |
| $\pi_{8}^{'}$ | 0.03 | 0.04 | 0.02 | 0.96 |  | 0.04 | 0.02 | 0.96 |  | 0.03 | 0.02 | 0.97 |
| $\pi_{9}^{'}$ | 0.01 | 0.02 | 0.02 | 0.95 |  | 0.02 | 0.02 | 0.95 |  | 0.02 | 0.01 | 0.95 |
| $\pi_{10}^{'}$ | 0.00 | - | - | - |  | 0.01 | 0.01 | 0.00 |  | 0.01 | 0.01 | 0.00 |
| $\pi_{11}^{'}$ | 0.00 | - | - | - |  | - | - | - |  | 0.01 | 0.01 | 0.00 |
| $\pi_{12}^{'}$ | 0.00 | - | - | - |  | - | - | - |  | 0.01 | 0.01 | 0.00 |
| $\pi_{13}^{'}$ | 0.00 | - | - | - |  | - | - | - |  | 0.01 | 0.01 | 0.00 |
| $\pi_{14}^{'}$ | 0.00 | - | - | - |  | - | - | - |  | 0.01 | 0.01 | 0.00 |
|  |  |  |  |  |  |  |  |  |  |  |  |  |
| DERIVED |  |  |  |  |  |  |  |  |  |  |  |  |
| $N_{1}$ | 160 | 164 | 16.53 | 0.96 |  | 166 | 17.21 | 0.95 |  | 173 | 21.11 | 0.89 |
| $N_{2}$ | 158 | 160 | 14.05 | 0.96 |  | 160 | 14.03 | 0.95 |  | 159 | 13.89 | 0.95 |
| $N_{3}$ | 156 | 158 | 11.95 | 0.97 |  | 158 | 11.96 | 0.97 |  | 158 | 12.02 | 0.97 |
| $N_{4}$ | 154 | 157 | 13.40 | 0.97 |  | 157 | 13.45 | 0.96 |  | 158 | 13.71 | 0.96 |
| $N_{5}$ | 152 | 157 | 15.48 | 0.96 |  | 157 | 15.67 | 0.96 |  | 159 | 16.32 | 0.96 |
| $N_{6}$ | 151 | 157 | 19.16 | 0.94 |  | 157 | 19.34 | 0.94 |  | 159 | 20.39 | 0.94 |
| $N_{7}$ | 149 | 157 | 22.72 | 0.96 |  | 158 | 23.07 | 0.94 |  | 160 | 24.37 | 0.93 |

Table S3. Detailed simulation results comparing expected values (mean), root mean squared error (RMSE), and credible coverage (Coverage) from 200 simulated data sets analyzed using age-structured Jolly-Seber models (AS-JS) where survival is a quadratic function of age. Data simulations set maximum age in year 1 ($J$) as 9, and we fit age-structured Jolly-Seber models assuming $J$ = 9, 10, or 14. Here, detection probability was increased of 0.50. See methods for complete parameter definitions and simulation settings.

|  |  | AS-JS ($J$ = 9) | | |  | AS-JS ($J$ = 10) | | |  | AS-JS ($J$ = 14) | | |
| --- | --- | --- | --- | --- | --- | --- | --- | --- | --- | --- | --- | --- |
| Parameter | TRUE | Mean | RMSE | Coverage |  | Mean | RMSE | Coverage |  | Mean | RMSE | Coverage |
| $N^{*}$ | 400 | 406 | 18.0 | 0.96 |  | 407 | 18.3 | 0.96 |  | 410 | 19.8 | 0.94 |
| $\alpha_{0}$ | 1.73 | 1.74 | 0.23 | 0.96 |  | 1.74 | 0.23 | 0.96 |  | 1.76 | 0.23 | 0.95 |
| $\alpha_{1}$ | -0.50 | -0.53 | 0.09 | 0.94 |  | -0.53 | 0.09 | 0.93 |  | -0.53 | 0.09 | 0.92 |
| $\alpha_{2}$ | -0.20 | -0.21 | 0.03 | 0.96 |  | -0.21 | 0.03 | 0.96 |  | -0.21 | 0.03 | 0.96 |
| $p$ | 0.50 | 0.49 | 0.02 | 0.96 |  | 0.49 | 0.02 | 0.96 |  | 0.49 | 0.02 | 0.94 |
| $\beta_{1}$ | 0.40 | 0.39 | 0.03 | 0.94 |  | 0.39 | 0.03 | 0.94 |  | 0.40 | 0.03 | 0.94 |
| $\beta_{2}$ | 0.10 | 0.10 | 0.02 | 0.96 |  | 0.10 | 0.02 | 0.96 |  | 0.10 | 0.02 | 0.96 |
| $\beta_{3}$ | 0.10 | 0.10 | 0.02 | 0.93 |  | 0.10 | 0.02 | 0.94 |  | 0.10 | 0.02 | 0.94 |
| $\beta_{4}$ | 0.10 | 0.10 | 0.02 | 0.96 |  | 0.10 | 0.02 | 0.96 |  | 0.10 | 0.02 | 0.95 |
| $\beta_{5}$ | 0.10 | 0.10 | 0.02 | 0.95 |  | 0.10 | 0.02 | 0.96 |  | 0.10 | 0.02 | 0.96 |
| $\beta_{6}$ | 0.10 | 0.10 | 0.02 | 0.96 |  | 0.10 | 0.02 | 0.96 |  | 0.10 | 0.02 | 0.96 |
| $\beta_{7}$ | 0.10 | 0.10 | 0.02 | 0.97 |  | 0.10 | 0.02 | 0.97 |  | 0.10 | 0.02 | 0.97 |
| $\pi_{1}^{'}$ | 0.27 | 0.26 | 0.04 | 0.96 |  | 0.25 | 0.04 | 0.94 |  | 0.24 | 0.05 | 0.88 |
| $\pi_{2}^{'}$ | 0.17 | 0.16 | 0.03 | 0.94 |  | 0.16 | 0.03 | 0.94 |  | 0.15 | 0.03 | 0.92 |
| $\pi_{3}^{'}$ | 0.14 | 0.14 | 0.03 | 0.94 |  | 0.13 | 0.03 | 0.93 |  | 0.13 | 0.03 | 0.90 |
| $\pi_{4}^{'}$ | 0.12 | 0.12 | 0.03 | 0.96 |  | 0.12 | 0.03 | 0.95 |  | 0.12 | 0.03 | 0.96 |
| $\pi_{5}^{'}$ | 0.11 | 0.11 | 0.02 | 0.96 |  | 0.10 | 0.02 | 0.96 |  | 0.10 | 0.03 | 0.96 |
| $\pi_{6}^{'}$ | 0.09 | 0.09 | 0.02 | 0.95 |  | 0.09 | 0.02 | 0.95 |  | 0.08 | 0.02 | 0.94 |
| $\pi_{7}^{'}$ | 0.06 | 0.06 | 0.02 | 0.97 |  | 0.06 | 0.02 | 0.97 |  | 0.06 | 0.02 | 0.96 |
| $\pi_{8}^{'}$ | 0.03 | 0.04 | 0.02 | 0.97 |  | 0.03 | 0.02 | 0.98 |  | 0.03 | 0.02 | 0.98 |
| $\pi_{9}^{'}$ | 0.01 | 0.02 | 0.01 | 0.95 |  | 0.02 | 0.01 | 0.95 |  | 0.01 | 0.01 | 0.95 |
| $\pi_{10}^{'}$ | 0.00 | - | - | - |  | 0.01 | 0.01 | 0.00 |  | 0.01 | 0.01 | 0.00 |
| $\pi_{11}^{'}$ | 0.00 | - | - | - |  | - | - | - |  | 0.01 | 0.01 | 0.00 |
| $\pi_{12}^{'}$ | 0.00 | - | - | - |  | - | - | - |  | 0.01 | 0.01 | 0.00 |
| $\pi_{13}^{'}$ | 0.00 | - | - | - |  | - | - | - |  | 0.01 | 0.01 | 0.00 |
| $\pi_{14}^{'}$ | 0.00 | - | - | - |  | - | - | - |  | 0.01 | 0.01 | 0.00 |
|  |  |  |  |  |  |  |  |  |  |  |  |  |
| DERIVED |  |  |  |  |  |  |  |  |  |  |  |  |
| $N_{1}$ | 160 | 161 | 8.1 | 0.94 |  | 162 | 8.3 | 0.94 |  | 164 | 9.5 | 0.92 |
| $N_{2}$ | 158 | 159 | 6.4 | 0.95 |  | 159 | 6.3 | 0.95 |  | 159 | 6.3 | 0.95 |
| $N_{3}$ | 156 | 157 | 6.3 | 0.97 |  | 157 | 6.3 | 0.96 |  | 157 | 6.3 | 0.97 |
| $N_{4}$ | 154 | 155 | 6.3 | 0.98 |  | 155 | 6.3 | 0.98 |  | 155 | 6.4 | 0.98 |
| $N_{5}$ | 152 | 154 | 7.1 | 0.96 |  | 154 | 7.1 | 0.96 |  | 154 | 7.2 | 0.96 |
| $N_{6}$ | 151 | 153 | 8.1 | 0.98 |  | 153 | 8.2 | 0.98 |  | 154 | 8.4 | 0.97 |
| $N_{7}$ | 149 | 152 | 10.5 | 0.97 |  | 153 | 10.6 | 0.96 |  | 153 | 10.9 | 0.96 |
